# Sensitization of KPC and NDM Klebsiella pneumoniae To Rifampicin by the Human Lactoferrin-Derived Peptide hLF1-11

**DOI:** 10.1128/spectrum.02767-22

**Published:** 2022-12-20

**Authors:** Paola Morici, Cosmeri Rizzato, Emilia Ghelardi, Gian Maria Rossolini, Antonella Lupetti

**Affiliations:** a Department of Translational Research and New Technologies in Medicine and Surgery, University of Pisa, Pisa, Italy; b Department of Experimental and Clinical Medicine, University of Florence, Florence, Italy; c Clinical Microbiology and Virology Unit, Florence Careggi University Hospital, Florence, Italy; University of Manitoba

**Keywords:** *Klebsiella pneumoniae*, KPC, NDM, antimicrobial peptides, lactoferrin-derived peptide, rifampicin

## Abstract

A synergistic effect of non-bactericidal concentrations of the human lactoferrin (hLF)-derived peptide hLF1-11 and rifampicin against multidrug-resistant KPC (Klebsiella pneumoniae carbapenemase)-producing K. pneumoniae has been previously shown. The present study focuses on the mechanism(s) underlying this synergistic effect. The contribution of hLF1-11 and rifampicin to the synergistic effect was evaluated by killing assays with KPC K. pneumoniae cells incubated with hLF1-11 and, after washing, with rifampicin, or *vice versa*. Cell membrane permeability and polarization upon exposure to hLF1-11 and/or rifampicin were evaluated by ethidium bromide (EtBr) and DiBAC_4_(3) (bis-1,3-dibutylbarbituric acid trimethine oxonol) permeability, respectively. The effect of carbonyl cyanide *m*-chlorophenyl hydrazone (CCCP), an uncoupler of oxidative phosphorylation, was also evaluated. KPC K. pneumoniae cells were effectively killed after prior exposure to rifampicin for 30 to 60 min followed by treatment with hLF1-11, while no antibacterial activity was observed when cells were incubated with hLF1-11 first and then with rifampicin. EtBr accumulation increased upon exposure to hLF1-11 or the combination of hLF1-11 and rifampicin, but not upon exposure to rifampicin alone. Moreover, hLF1-11 induced a dose-dependent membrane depolarization. As expected, the antibacterial activity of hLF1-11 alone or combined with rifampicin was significantly reduced in the presence of CCCP. Furthermore, hLF1-11 and rifampicin were synergistic also against a colistin-resistant NDM (New Delhi metallo-β-lactamase)-producing K. pneumoniae strain. The results suggest that rifampicin was accumulated by KPC cells during the 30-to-60-min incubation and that the addition of hLF1-11 sensitized bacterial cells to rifampicin by inducing a transient loss of membrane potential and increased cell membrane permeability, thus facilitating the entrance and retention of rifampicin into the cytoplasm.

**IMPORTANCE** The present study describes a synergistic effect between rifampicin, an impermeable hydrophobic antibiotic with an intracellular target, and an hLF1-11, an antimicrobial peptide derived from human lactoferrin, against multidrug-resistant Klebsiella pneumoniae. Carbapenem-resistant K. pneumoniae has recently caused an outbreak in Tuscany, Italy, thus pressing the need for the development of new treatment options. The mechanisms underlying such a synergistic effect have been studied. The results suggest that the synergistic effect was due to the transient loss of membrane potential induced by hLF1-11 and the subsequent increase in cell membrane permeability which allowed rifampicin to enter the bacterial cell. Therefore, it is likely that a sub-inhibitory concentration of hLF1-11 can efficiently permeabilize K. pneumoniae cells to rifampicin, allowing the antibiotic to reach its intracellular target. These results encourage further exploration of possible applications of this synergistic combination in the treatment of K. pneumoniae infections.

## INTRODUCTION

Carbapenem-resistant Enterobacterales (CRE) are associated with significant morbidity and mortality, representing a growing threat to public health worldwide. They have been recognized as one of the three most urgent antimicrobial-resistant threats by the CDC and as pathogens of critical priority by the World Health Organization (WHO) (1). Since 2010, an epidemic spread has been observed in Italy, with K. pneumoniae producing KPC-type carbapenemases (KPC-KP), mostly from Clonal Group 258, representing the majority of strains (2, 3). In late 2018, in Tuscany, New Delhi metallo-β-lactamase-producing carbapenem-resistant Enterobacterales (NDM-CRE) caused an outbreak in which K. pneumoniae ST147/NDM-1 was the dominant clone (4, 5).

The spread of carbapenem-resistant K. pneumoniae has caused a pressing need for the development of new treatment options for these infections. Although several new antibiotics effective against CRE have recently been introduced in clinical practice, they mostly target KPC-KP, while coverage of strains producing other carbapenemases and especially metallo-β-lactamases (MBLs) remains limited (6). In this perspective, treatments based on the use of various antibiotic combinations have been suggested (7) and, in this context, antimicrobial peptides are considered promising therapeutic agents, exerting synergistic effects when tested *in vitro* in combination with conventional antibiotics (8–12).

A synthetic peptide comprising the first cationic domain of human lactoferrin (hLF), here referred to as hLF1-11, possesses high antibacterial activity, as demonstrated by *in vitro* and *in vivo* studies in systemic infections caused by multidrug-resistant Staphylococcus aureus and Acinetobacter baumannii strains (13, 14). The potent antimicrobial effect of hLF1-11 can be attributed to its positive charge and hydrogen-binding properties which allow interaction with negatively charged components of the bacterial cell wall (11, 15). Recently, it has been demonstrated that hLF1-11 alone or combined with conventional antibiotics, especially hydrophobic antibiotics, is highly effective against K. pneumoniae clinical isolates harboring different resistant genes (16). This study was performed prior to the emergence of NDM-CRE.

The mechanisms which lead to the synergistic effect of hLF1-11 with hydrophobic antibiotics such as rifampicin are uncertain. Bacterial membrane depolarization and increased permeability, which are killing mechanisms exerted by various antimicrobial peptides, may be involved in such synergistic effects, possibly allowing intracellular antibiotic concentrations to reach lethal levels (17–20).

The present study was undertaken (i) to evaluate whether the combination of hLF1-11 and rifampicin was also synergistic against a selected colistin-resistant NDM K. pneumoniae strain and (ii) to gain more insight into the molecular mechanisms underlying the synergistic antibacterial effect induced by the same combination against a previously characterized colistin-resistant clinical isolate of KPC-producing K. pneumoniae (16).

## RESULTS

Antimicrobial therapies combining conventional antibiotics with antimicrobial peptides have been proposed for the treatment of multidrug-resistant infections, especially those caused by Gram-negative bacteria (21). Although most Gram-negative bacteria are intrinsically resistant to rifampicin, this antibiotic has already been adopted in combination therapies for the treatment of multidrug-resistant enterobacteria, such as KPC strains (22, 23). Furthermore, previous studies have demonstrated that the capsule can inhibit the effect of antimicrobial peptides by electrostatic and hydrophobic interactions which prevent them from reaching the bacterial membrane (24, 25). Nevertheless, hLF1-11 seems to retain its activity against encapsulated K. pneumoniae.

Therefore, synergy studies combining various concentrations of hLF1-11 with rifampicin against a colistin-resistant NDM K. pneumoniae strain were performed using the checkerboard method. The results revealed that the hLF1-11 peptide exerted a synergistic effect in combination with rifampicin against the colistin-resistant NDM K. pneumoniae isolate (Table 1), similarly to previously tested K. pneumoniae strains (16). Indeed, in the presence of hLF1-11, the MIC of rifampicin decreased from 128 μg/mL to 8 μg/mL.

**TABLE 1:** MIC values of hLF1-11 or rifampicin and the synergistic effect of their combination, expressed as FIC index, against NDM Klebsiella pneumoniae strain and K. pneumoniae 1R^*a*^

Strain	MIC (μg/mL)^*b*^		FIC index^*c*^
	hLF1-11	Rifampicin	
KPC K. pneumoniae 1R^*d*^	44	16	0.35
NDM K. pneumoniae	176	128	0.15

a FIC, fractional inhibitory concentration; NDM, New Delhi metallo-β-lactamase-producing; KPC, Klebsiella pneumoniae carbapenemase-producing.

b MIC values were obtained by microdilution method in 1:16 diluted Mueller-Hinton broth.

c Mean of the lowest FIC indices of at least three independent experiments. Mean FIC value of ≤0.5 indicates synergism.

d Previously published by Morici et al. (16).

### Synergistic effect between hLF1-11 and rifampicin against the colistin-resistant KPC *K. pneumoniae* 1R strain is bactericidal.

**FIG 1:**
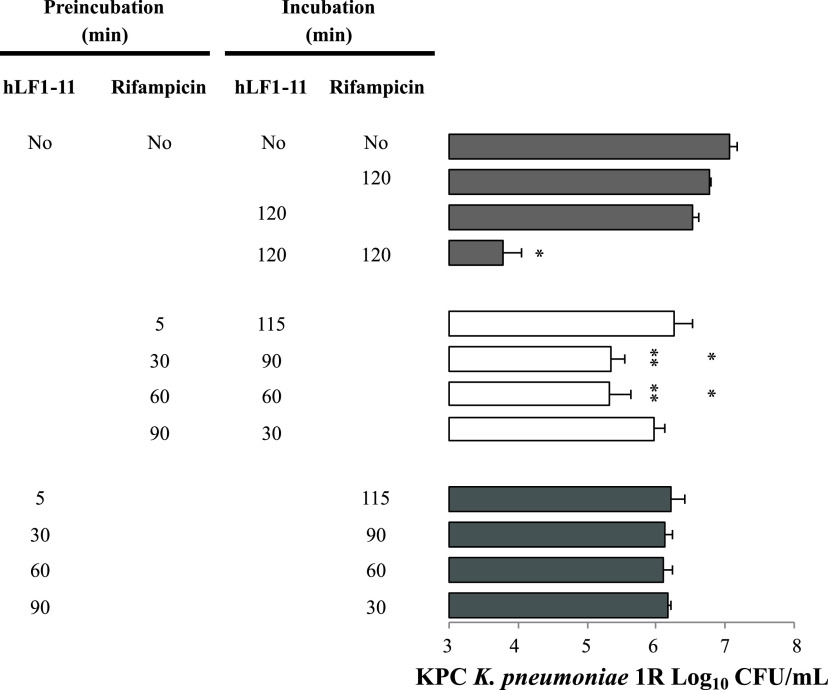
Effect of preincubation of hLF1-11 and/or rifampicin on the synergistic effect of the combination against Klebsiella pneumoniae carbapenemase (KPC)-producing K. pneumoniae 1R strain. Bacterial cells (10^6^ CFU/mL) were incubated for 5, 30, 60, and 90 min with 22 μg/mL hLF1-11 or 4 μg/mL rifampicin, washed, and incubated for 115, 90, 60 and 30 min with the same concentrations of rifampicin or hLF1-11, respectively. Data are expressed as the means ± standard deviation (SD) of three independent experiments. *, *P* < 0.05 compared to KPC K. pneumoniae 1R exposed to hLF1-11 or rifampicin alone. **, *P* < 0.05 compared to KPC K. pneumoniae 1R exposed for 2 h to the combination of hLF1-11 with rifampicin. “No,” no agent.

To assess whether hLF1-11 and rifampicin act synergistically to kill a colistin-resistant K. pneumoniae strain, we performed bacterial killing assays using non-bactericidal concentrations of the antimicrobial peptide and antibiotic. Our results revealed that the combination of non-bactericidal concentrations of hLF1-11 (22 μg/mL) and rifampicin (4 μg/mL) for 1 h at 37°C exerted a synergistic effect, reaching more than 2-log reduction compared to the most effective compound, against the colistin-resistant KPC K. pneumoniae 1R strain (Fig. 1).

In addition, data obtained from the minimum bactericidal concentration (MBC) determination for K. pneumoniae 1R strain confirmed the bactericidal effect of the synergistic activity after 24 h of incubation. MBCs of hLF1-11 and rifampicin were similar to their MICs (hLF1-11, 22 to 44 μg/mL; rifampicin, 32 μg/mL). In the presence of hLF1-11 (5.5 μg/mL), the MBC of rifampicin was 4-fold reduced (from 32 to 8 μg/mL), with a log CFU reduction of 4.3 compared to the initial inoculum. These results show that the synergistic activity of hLF1-11 and rifampicin is bactericidal.

### Effect of pre-incubation with non-bactericidal concentrations of hLF1-11 or rifampicin on the bactericidal activity of rifampicin or hLF1-11.

To gain more insight into the molecular mechanisms underlying the synergistic effect between hLF1-11 and rifampicin, we investigated whether subsequent exposure of bacteria to the two compounds individually yielded a synergistic effect as observed for the 2-h contemporary exposure. To this end, K. pneumoniae 1R cells were treated for 2 h with a combination of the two compounds using the following scheme: cells were pre-incubated for 5, 30, 60, or 90 min with a non-bactericidal concentration of rifampicin (4 μg/mL) and, after washing, incubated with a non-bactericidal concentration of hLF1-11 (22 μg/mL) for as many minutes as needed to reach 2 h; or, *vice versa*, a pre-incubation with hLF1-11 for 5, 30, 60, or 90 min followed by an incubation with rifampicin.

The results revealed that the antibacterial activity obtained after pre-incubation with rifampicin for 30 or 60 min followed, after washing, by incubation with hLF1-11 reached about a 1.5-log reduction (*P* < 0.05) in CFU/mL compared to untreated cells (Fig. 1). However, this antibacterial effect was significantly (*P* < 0.05) lower than that obtained by co-incubation of hLF1-11 and rifampicin for 2 h.

In contrast, when K. pneumoniae cells were pre-incubated with hLF1-11 followed by rifampicin, for all combinations of incubation times, no bactericidal effect was observed, suggesting a temporary effect of the peptide.

Another interesting observation was that no antibacterial effect was observed when cells were pre-incubated with rifampicin for 5 or 90 min before exposure to hLF1-11 for 115 or 30 min.

Because the outer membrane of *Enterobacteriaceae* represents an impermeable barrier for rifampicin (26), these results suggest that rifampicin (slowly) adhered to the extracellular surface of the outer membrane of K. pneumoniae, was retained during cell washing, and was subsequently internalized into the bacterial cell after outer membrane permeabilization by hLF1-11. This is consistent with previous evidence showing that several antimicrobial peptides can permeabilize the outer membrane of Gram-negative bacteria (21, 27–29).

The observation that a 90-min incubation with rifampicin followed by 30 min with hLF1-11 did not significantly reduce the viable cell count suggests that more than 30 min is required for rifampicin to reach its target and exert an effect.

### Kinetics of synergistic activity.

**FIG 2:**
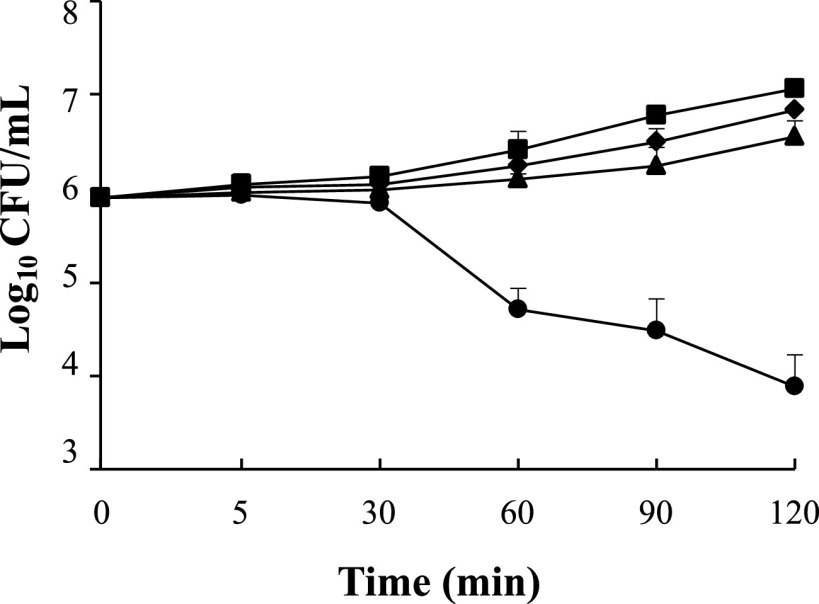
Time-kill curves of hLF1-11 in combination with rifampicin against KPC K. pneumoniae 1R strain. K. pneumoniae cells (10^6^ CFU/mL) were incubated with 22 μg/mL hLF1-11 (triangle), 4 μg/mL rifampicin (diamond), the combination of the same concentrations of hLF1-11 and rifampicin (circle), or no treatment (square) until 120 min. The viable cell count was determined at each time point. Data are expressed as the means ± SD of three independent experiments.

Based on the previously mentioned observation regarding the lack of antibacterial activity shown by pre-incubation with rifampicin for 90 min followed by incubation with hLF1-11 for 30 min, we investigated the killing kinetics of the synergistic effect. The results revealed a significant (*P* < 0.05) antibacterial effect at 60 min, and a synergistic effect at 2 h of co-incubation of the two compounds against colistin-resistant K. pneumoniae cells (Fig. 2).

### Effect of hLF1-11 on cell permeability.

We hypothesized that the subsequent addition of hLF1-11 could transiently alter membrane permeability and potential, facilitating the entrance of rifampicin into the cytoplasm.

Therefore, to gain more insights into the role of hLF1-11 in this synergistic effect, we measured the effect of hLF1-11 on cell permeability using an ethidium bromide (EtBr) permeability assay.

EtBr accumulation in the cell is the result of an interplay between cell membrane permeability and efflux pumps, which are dependent on the proton motive force (PMF) (30). Once inside the bacterial cell, EtBr intercalates between the nitrogenous bases of DNA, enhancing its fluorescence intensity compared to when it is free in aqueous solution. The binding constant is strong enough to keep EtBr inside cells, avoiding the efflux pump system (31).

**FIG 3:**
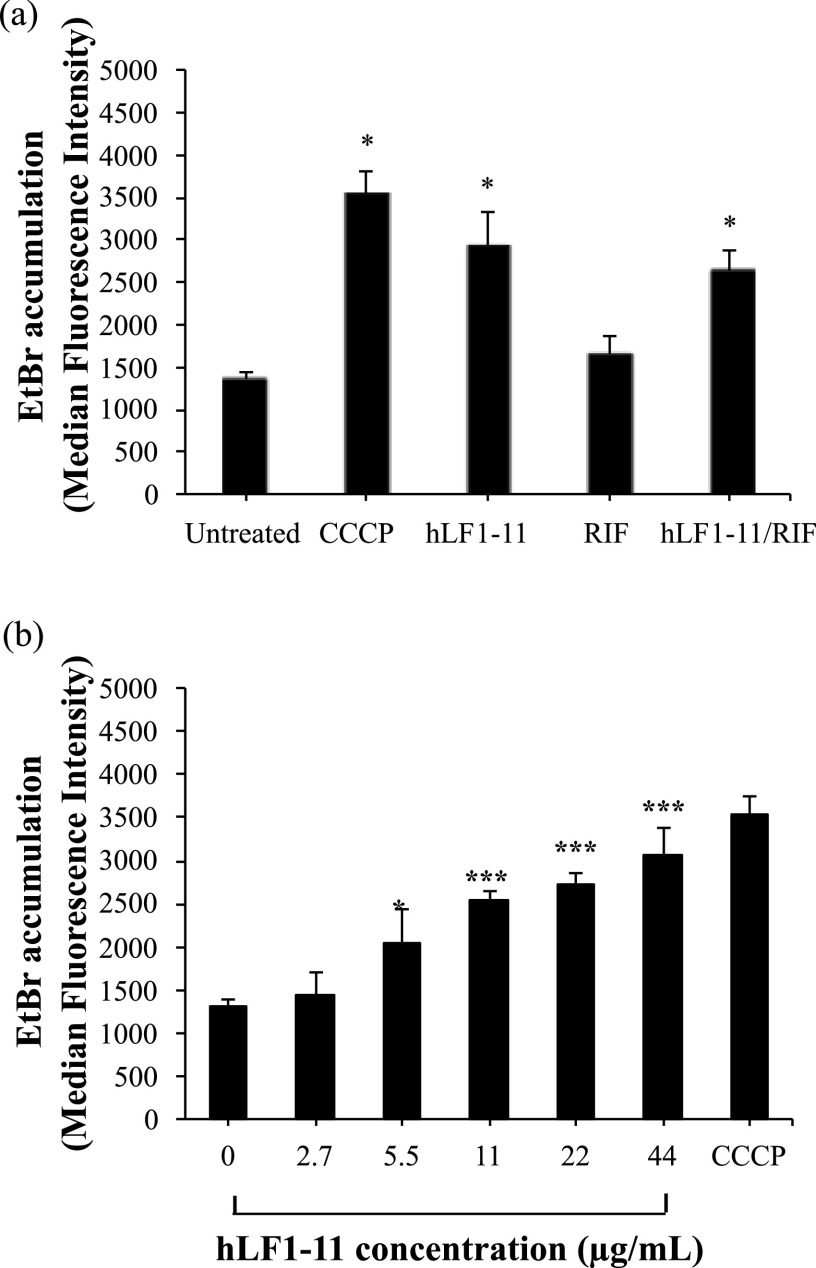
Ethidium bromide (EtBr) accumulation induced by hLF1-11, alone and in combination with rifampicin. KPC K. pneumoniae 1R cells (10^7^ CFU/mL) were exposed to (a) subinhibitory concentrations of hLF1-11 (22 μg/mL) and/or rifampicin (4 μg/mL), and (b) various concentrations of hLF1-11, at 37°C for 1 h at the dark, and then fixed in 1% paraformaldehyde. Before measuring the EtBr fluorescence, fixed cells were recovered and resuspended in phosphate-buffered saline. Fluorescence emission was detected by a BD Accuri C6 flow cytometer in the FL2 channel. Positive control represents bacteria untreated and incubated with CCCP (carbonyl cyanide *m*-chlorophenyl hydrazone, 100 μM) and EtBr (1 μg/mL). Data are expressed as the means ± SD of three independent experiments. *, *P* < 0.05; ***, *P* < 0.001 compared to untreated KPC K. pneumoniae 1R cells.

To evaluate the effect of hLF1-11 and/or rifampicin on the permeability of bacterial cells to EtBr, we used flow cytometry to measure the fluorescence of intracellular EtBr after cell exposure to the antimicrobial peptide and/or rifampicin. Because CCCP (carbonyl cyanide *m*-chlorophenyl hydrazone) is an uncoupler of oxidative phosphorylation, we used it to dissipate the PMF. As shown in Fig. 3, the inhibition of the efflux pumps allowed EtBr accumulation inside bacterial cells, thus resulting in a significant fluorescence (*P* < 0.001) increase compared to that in cells not exposed to CCCP. The flow cytometric analysis revealed that both cells treated with hLF1-11 (22 μg/mL) and cells treated with the hLF1-11/rifampicin combination displayed significantly (*P* < 0.001) increased EtBr fluorescence intensity compared to untreated cells (Fig. 3a). No increase in EtBr fluorescence intensity was induced by exposure to rifampicin alone (4 μg/mL). Furthermore, the hLF1-11 peptide induced an increase in EtBr fluorescence intensity in a dose-dependent manner (Fig. 3b).

These results support our hypothesis that hLF1-11 facilitates the entrance of rifampicin into K. pneumoniae cells.

### Effect of CCCP on the hLF1-11 antibacterial activity.

Since it has been previously hypothesized that hLF1-11 requires energized cells for its antifungal activity (32, 33), we evaluated the effect of CCCP, an uncoupler of oxidative phosphorylation used to dissipate the PMF, on the bactericidal activity of hLF1-11 and rifampicin.

**FIG 4:**
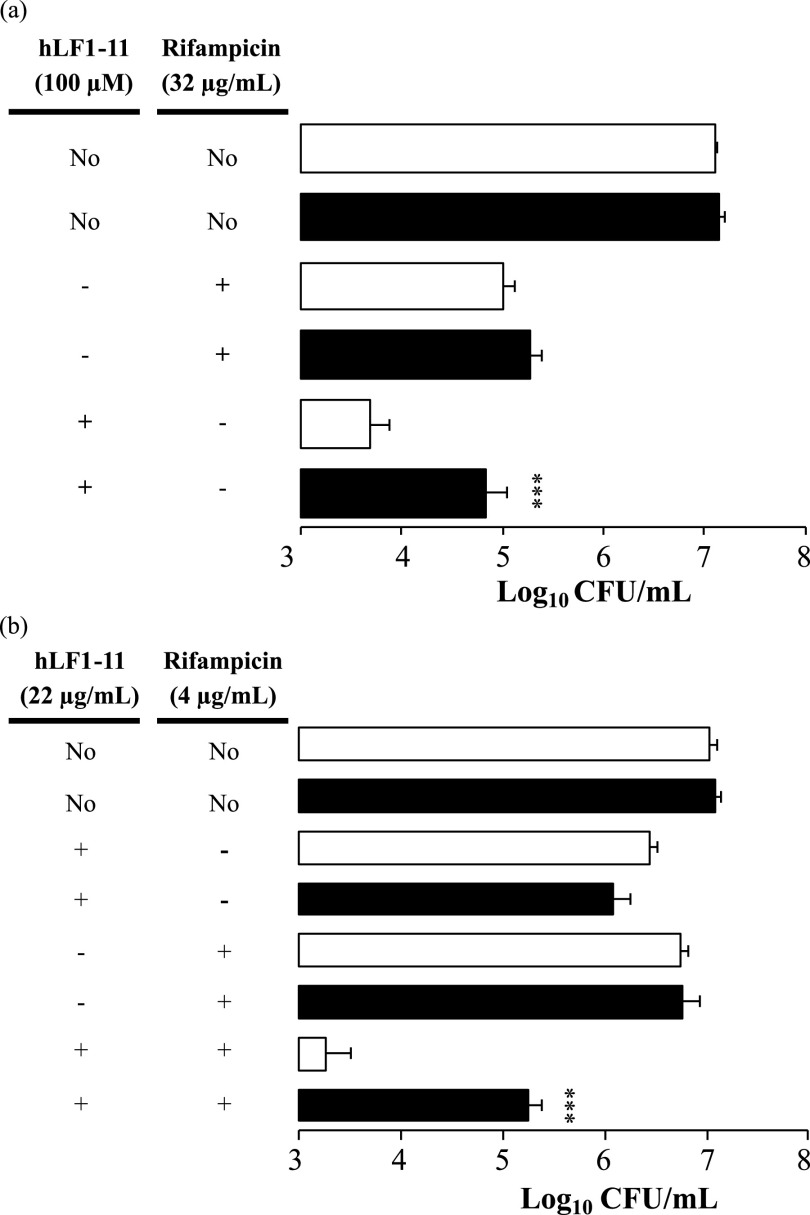
Effect of CCCP on the antibacterial activity of hLF1-11 alone and in combination with rifampicin. KPC K. pneumoniae cells (10^6^ CFU/mL) were incubated in the presence (closed bars) or absence (open bars) of 100 μM CCCP for 10 min at 37°C prior to treatment with (a) bactericidal concentrations of hLF1-11 (100 μM) or rifampicin (32 μg/mL), or sub-inhibitory concentrations of hLF1-11 (22 μg/mL) and/or rifampicin (4 μg/mL). After incubation at 37°C for 1 h, viable cell counts were carried out. Data are expressed as the means ± SD of three independent experiments. ***, *P* < 0.001 compared to KPC K. pneumoniae cells exposed to (a) hLF1-11 (100 μM) or (b) a combination of hLF1-11/rifampicin, in the presence or absence of CCCP.

To this purpose, K. pneumoniae cells were first exposed to CCCP and then treated with a bactericidal concentration of hLF1-11 or rifampicin. The results revealed that the bactericidal effect of hLF1-11 (100 μM) was significantly (*P* < 0.001) decreased by previous exposure to CCCP (Fig. 4a). No inhibitory effect of CCCP was observed on the bactericidal activity of rifampicin. Next, we evaluated the inhibitory effect of CCCP on the synergism induced by the combination of sub-inhibitory concentrations of hLF1-11 and rifampicin (Fig. 4b). The results revealed that CCCP significantly (*P* < 0.001) reduced the synergistic effect induced by the combination of hLF1-11 with rifampicin.

These results show that dissipation of the PMF decreases the bactericidal activity of hLF1-11, both alone and in combination with rifampicin.

### Cytoplasmic membrane depolarization.

**FIG 5:**
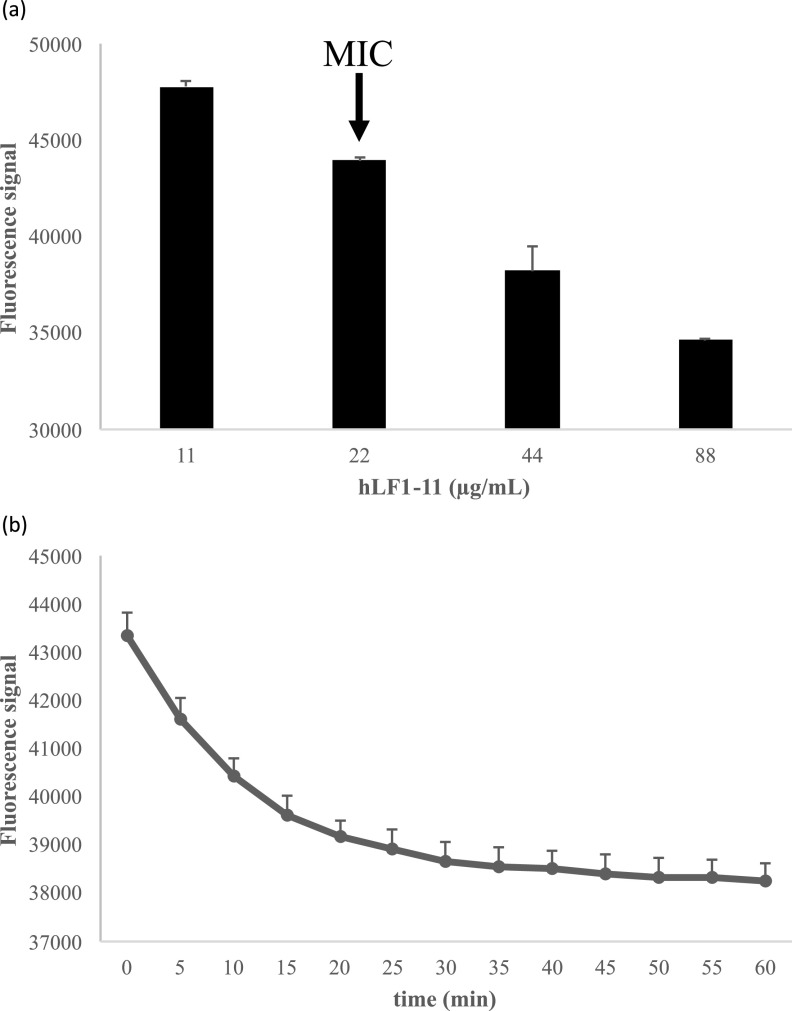
Cytoplasmic membrane depolarization induced by the hLF1-11 peptide. A suspension of K. pneumoniae 1R cells (10^7^ CFU/mL) in mid-log-phase growth was incubated with (a) various concentrations of hLF1-11 for 1 h a 37°C or (b) 2× MIC hLF1-11 to assess the membrane depolarization at earlier time points (up to 1 h). DiBAC_4_(3) (bis-1,3-dibutylbarbituric acid trimethine oxonol) fluorescence was measured in real time. Antimicrobial activity is shown as decreased fluorescence while DiBAC_4_(3) is internalized in depolarized bacteria. Data are expressed as the means ± standard error of three independent experiments.

To evaluate whether hLF1-11 was able to induce cytoplasmic membrane depolarization, we used the anionic dye DiBAC_4_(3), a lipophilic membrane potential-sensitive dye with a low binding affinity for intact membranes. This dye accumulates inside depolarized cells by binding to intracellular proteins and membranes, thus resulting in a decrease of fluorescence signal in the medium. Our results revealed that hLF1-11 induced cytoplasmic membrane depolarization in a dose-dependent manner (Fig. 5a). Other studies have described similar evidence showing a concentration-dependent depolarizing effect of the peptide LL-37 on Pseudomonas aeruginosa and PMAP-36, GI24, and melittin on Escherichia coli cell membrane (34), thus suggesting membrane depolarization as a prominent effect of peptide-membrane interactions (35). On the contrary, other peptides known to carry out their antibacterial activity through intracellular targets do not induce membrane permeability and depolarization (36, 37).

To shed light onto the molecular basis of these effects, we evaluated the timing of hLF1-11 induced membrane depolarization at 2× MIC. Our results showed that, at early time points, cytoplasmic membrane depolarization induced a 40% decrease in medium fluorescence, reaching a plateau after 35 min (Fig. 5b).

Interestingly, at sub-inhibitory concentrations, cell viability was maintained, as shown by killing assays, but the cytoplasmic membrane was transiently depolarized, as shown by decreased DiBAC_4_(3) fluorescence.

Overall, these results show that exposure to hLF1-11 was able to induce both cell membrane permeabilization and membrane depolarization, consistent with our hypothesis.

### Conclusions.

In conclusion, the overall data suggest that sensitization of KPC K. pneumoniae 1R to rifampicin, an impermeable hydrophobic antibiotic, may be due to the transient loss of membrane potential induced by hLF1-11 and the subsequent increase in cell membrane permeability, while cell viability is maintained. Therefore, it is likely that a sub-inhibitory concentration of hLF1-11 can efficiently permeabilize K. pneumoniae cells to rifampicin, allowing the antibiotic to reach its intracellular target. Furthermore, by destabilizing K. pneumoniae membrane, all membrane-associated functions may be hampered, making hLF1-11 a potential strategy to enhance many inactive antibiotics against these multidrug-resistant microorganisms. In a previous study (16), we demonstrated synergistic effects of hLF1-11 and other antibiotics, such as clarithromycin, clindamycin, and gentamicin, against carbapenemase-producing K. pneumoniae strains harboring different resistance genes.

To our knowledge, clinical evidence to support the use of combined therapeutic regimens with rifampicin is still lacking and needs to be assessed (38). Our results encourage further exploration of possible applications of this synergistic combination in the treatment of CRE infections.

A deeper understanding of the therapeutic efficacy, safety, and tolerability of this synergistic interaction in *in vivo* animal models and clinical settings remains to be achieved.

## MATERIALS AND METHODS

### Bacterial strain and growth conditions.

Two K. pneumoniae strains were used in this study: one colistin-resistant (MIC = 16 μg/mL) and harboring the *bla*_KPC-3_ gene (strain 1R), isolated from a patient admitted to the Azienda Ospedaliero-Universitaria Pisana (Pisa, Italy) (16), and one colistin-resistant (MIC = 32 μg/mL) isolated from a patient admitted to the Ospedale San Luca di Lucca (Lucca, Italy) during the Tuscan NDM-CRE outbreak and belonging to the ST147 clonal lineage harboring the *bla*_NDM_ gene.

Bacteria were cultured in Luria Bertani (LB) broth (Sigma-Aldrich, St. Louis, MO, USA) to mid-log-phase. Aliquots of this culture were supplemented with 20% (vol/vol) glycerol and stored at −80°C. Prior to each experiment, bacterial strains were cultured overnight in LB broth at 37°C and subcultured for 2 h (mid-log-phase) under aerobic conditions at 37°C.

### Synthetic peptide and antibiotic.

The synthetic peptide corresponding to residues 1 to 11 of human lactoferrin, named hLF1-11 (GRRRRSVQWCA; molecular mass = 1,374.6 Da), was purchased from Peptisyntha (Brussels, Belgium). The purity of the hLF1-11 peptide exceeded 99%, as determined by reversed-phase high-performance liquid chromatography (RP-HPLC). Stocks of the peptide at a concentration of 10 mM in 0.1% acetic acid (pH 3.7) were stored at −20°C and diluted to the desired concentrations before use. Rifampicin (Sigma-Aldrich) was dissolved at 20 mg/mL in dimethyl sulfoxide (DMSO; Fluka Chemie GmbH, Sigma-Aldrich Chemie BV, Zwijndrecht, The Netherlands) and stored at −80°C. The final concentration of DMSO was <0.1% in all assays.

### Synergy studies.

Synergy analyses of hLF1-11 and rifampicin were carried out by a checkerboard titration method using 96-well round-bottomed polystyrene microtiter plates. This assay was performed as previously described (16) in Mueller-Hinton broth (MHB; Oxoid, Milan, Italy) diluted 1/16 in 10 mM Na-phosphate buffer (NaPB [pH 7.4]) because hLF1-11 was less effective in full-strength medium.

The concentration ranges tested for rifampicin and hLF1-11 peptide were 0.125 to 32 μg/mL and 2.7 to 352 μg/mL, respectively.

After 18 to 24 h of incubation at 37°C, the MICs of both the peptide and rifampicin were defined, based on the turbidity of the wells, as the lowest concentrations of the agent that completely inhibited visible growth. A variability of one dilution was considered acceptable to determine the MICs of hLF1-11 and rifampicin for the NDM-CRE strain tested. The fractional inhibitory concentration (FIC) index for the combinations was calculated using the following formula: FIC index = (MIC drug A in combination)/(MIC drug A alone) + (MIC drug B in combination)/(MIC drug B alone). The FIC indices were interpreted as follows: <0.5, synergy; 1 to 4, indifference; and >4, antagonism (39). The FIC index was reported in this study as the mean of the lowest FIC indices from at least three independent experiments.

### Determination of MBC.

The MBCs of hLF1-11 and rifampicin were determined for K. pneumoniae 1R strain using a checkerboard assay. After 24 h of incubation, viable cell counts (CFU/mL) were determined from wells where visible growth was inhibited (i.e., those at or above the MIC) and from control wells by plating serial dilutions of each sample on blood agar plates. MBC was defined as a log bacterial reduction of ≥3 compared to the initial inoculum (40, 41). Next, viable cells were determined in each well of the hLF1-11/rifampicin combination where FIC index was <0.5. All assays were performed in triplicate.

### Exposure of KPC *K. pneumoniae* 1R to hLF1-11 and/or rifampicin.

To gain a deeper insight into the contributions of the different agents to the synergistic effect of hLF1-11 peptide and rifampicin, bacterial cells in the mid-log-phase were harvested by centrifugation (4,500 × *g*, 10 min), washed twice with NaPB, and suspended at 10^7^ cells/mL in the same buffer. The bacterial suspension was mixed with equal volumes of sub-inhibitory concentrations of hLF1-11 or rifampicin and pre-incubated at 37°C for 5, 30, 60, and 90 min in 1/16 strength MHB diluted in NaPB. At each time point, cells were washed in NaPB at 4°C and then incubated with the other antimicrobial agent until reaching a total 2 h of incubation at 37°C. For such an experiment, the incubation time was 1 h longer to compensate for reduced exposure due to the removal of one of the two compounds. The number of viable cells was determined by plating serial dilutions of each sample on blood agar plates. For each combination of antimicrobial drug/exposure time, at least three independent experiments were carried out.

### Time-kill assay of the synergistic combination.

Time-kill synergy studies were performed by incubating K. pneumoniae cells (10^6^ CFU/mL) with sub-inhibitory concentrations of rifampicin (4 μg/mL, 1/4× MIC) and/or hLF1-11 (22 μg/mL, 1/2× MIC) in 1/16 diluted MHB. At each time point (0, 5, 30, 60, and 120 min), viable cell counts were performed as previously described. Synergy was defined as a decrease in CFU/mL of ≥ 2 log between the combination of hLF1-11 and antibiotic and its most active constituent (42). All assays were performed in triplicate.

### Ethidium bromide permeability assay.

KPC K. pneumoniae 1R cells (10^7^ CFU/mL) were mixed with various concentrations of the hLF1-11 peptide and/or rifampicin and incubated with 1 μg/mL EtBr in diluted MHB at 37°C for 1 h in the dark. After incubation, samples were centrifuged (4,500 × *g*, 5 min), washed with NaPB, and fixed in 1 mL of 1% paraformaldehyde (Sigma-Aldrich) at room temperature for 10 min and then overnight at 4°C. As a positive control, bacterial cells were incubated with 100 μM CCCP (Sigma-Aldrich), which inhibits proton motive force-dependent processes, including efflux pumps. Untreated cells and cells treated with EtBr alone were used as negative controls. Before the EtBr fluorescence was measured, cells were collected by centrifugation and resuspended in 300 μL phosphate-buffered saline. The emission of EtBr fluorescence was detected using a BD Accuri C6 flow cytometer (BD Biosciences, San Jose, CA, USA) equipped with a 488 nm laser and a 585/40 optical filter (FL2 channel). For each sample, at least 5,000 events were acquired and analyzed using the BD Accuri C6 software.

### Effect of CCCP on the antibacterial activity of hLF1-11.

The influence of proton motive force on the antibacterial activity of hLF1-11 was determined by killing assays in the presence of the metabolic uncoupler CCCP. A stock solution of 10 mM CCCP was prepared in water. KPC K. pneumoniae 1R cells were pretreated with 100 μM CCCP for 10 min at 37°C prior to the addition of hLF1-11 and/or rifampicin. After incubation for 1 h at 37°C in 1:16 diluted MHB, cell viability was assessed microbiologically.

### Cytoplasmic membrane depolarization.

Bacterial cells (10^7^ CFU/mL), harvested in mid-log-phase, were incubated in 1:16 diluted MHB with various concentrations of hLF1-11 for 1 h at 37°C. At the same time, samples were stained with 10 μg/mL DiBAC_4_(3) (Sigma-Aldrich) added from a stock solution of 5 mg/mL in DMSO. The hLF1-11 peptide was added at 44 μg/mL (2× MIC) to assess membrane depolarization at different time points for up to 1 h. Untreated cells were used as the negative control, and CCCP as the positive control. The fluorescence emission of DiBAC_4_(3) was detected as green fluorescence using 485 excitation/520 emission optical filters on a FLUOstar OPTIMA Microplate Reader (BMG LABTECH, Ortenberg, Germany). The depolarizing activity of the hLF1-11 peptide was evaluated as decreased fluorescence in the medium after uptake of DiBAC_4_(3).

### Statistical analysis.

Results were evaluated by a one-way analysis of variance test. Differences between the results of the various treatments were evaluated with a Tukey-Kramer test. The significance threshold was set at a *P* value of 0.05.

### Data availability.

All data are presented in the article.
